# Orthogonal conjugation of anchoring-dependent membrane active peptides for tuning of liposome permeability†

**DOI:** 10.1039/d5tb00304k

**Published:** 2025-07-10

**Authors:** Alexandra Iversen, Johanna Utterström, Basab Kanti Das, Robert Selegård, Lalit Pramod Khare, Daniel Aili

**Affiliations:** a Laboratory of Molecular Materials, Division of Biophysics and Bioengineering, Linköping University 581 83 Linköping Sweden daniel.aili@liu.se

## Abstract

Liposomes are widely utilized in drug delivery systems to enhance pharmacokinetics and reduce side effects. Bioresponsive membrane-active peptides (MAPs) can modulate the release of encapsulated drugs from liposomes, improving therapeutic efficacy. However, achieving efficient and specific conjugation of MAPs to liposomes remains challenging, complicating translational efforts. Thiol–maleimide Michael addition is an attractive strategy for lipid conjugation of cysteine-containing MAPs but is hindered by thiol oxidation. Strain-promoted azide–alkyne cycloaddition (SPAAC) presents an alternative conjugation approach, yet its effects on liposome and peptide functionality are not fully understood. Here, we demonstrate how these two conjugation strategies influence peptide–liposome interactions and lipid membrane permeability using a *de novo* designed bioresponsive MAP. Both strategies result in MAP accumulation on liposomes, but their effects on membrane integrity and release dynamics differ significantly. SPAAC-based conjugation is generally much slower than the corresponding thiol–maleimide reaction. The inclusion of cholesterol in liposomes has a pronounced impact on both conjugation reactions, leading to phase separation and unexpected cross-reactivity. Accounting for these effects enabled orthogonal MAP–liposome functionalization and selective, sequential release from mixed liposome populations using different MAPs. These findings highlight the critical role of conjugation chemistry and lipid composition in designing MAP-based bioresponsive liposomal drug delivery systems. Understanding these interactions allows for the fine-tuning of liposomal formulations to optimize drug release, opening new avenues for enhancing the efficacy of liposomal therapeutics.

## Abstract 2

Conjugation of designed membrane-active peptides to liposomes *via* thiol–maleimide and DBCO-based copper-free click chemistry enables triggered release, revealing lipid composition effects on conjugation specificity and release kinetics.

## Graphical abstract

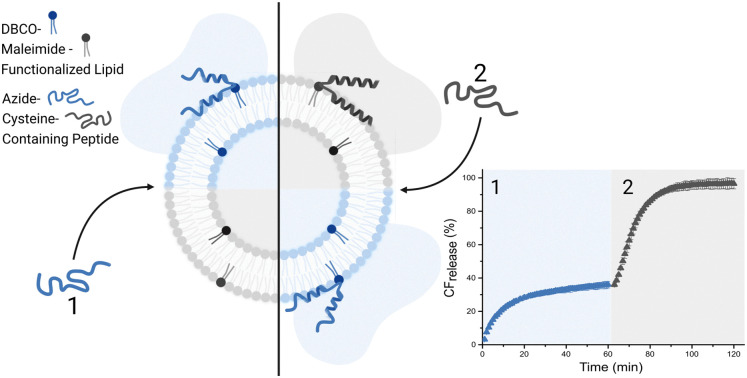

## Introduction

Liposomes are lipid vesicles that can be designed to carry a wide range of different drugs. The drugs can be incorporated either in the aqueous core, in the hydrophobic lipid bilayer, or tethered to the lipid membrane, thus providing a versatile platform for delivery of a wide range of different compounds.^1^ Liposome-based drug delivery systems (DDSs) can demonstrate long circulation times and reduce undesired side effects of the drugs^1^ by improving drug biodistribution, pharmacokinetics and dynamics.^2^ However, to become bioavailable the drugs must be released, which can occur by passive diffusion, liposomal degradation, or cellular uptake of the liposomes.^3^ The limited possibilities to tailor the drug release process can reduce therapeutic efficacy of liposomal drug formulations.^4^ The use of membrane active peptides (MAP) have been investigated as a strategy for modulating the release characteristics of liposome encapsulated drugs.^5–7^ MAPs are a diverse group of peptides, including natural and synthetic antimicrobial peptides (AMPs) and cell-penetrating peptides (CPPs), which interact with lipid membranes and affect their structure and function.^8^ AMPs and CPPs can have significant therapeutic potential as antimicrobial agents,^9^ as components in drug delivery vehicles,^10^ and by modulating cell membrane interactions for treatment of various diseases.^11^ MAPs are typically not very selective but can be modified to allow for specific and triggered liposomal release. For example, Sun *et al.* used a gain-of-function evolution strategy to identify MAPs based on the AMP melittin that were specific for PEGylated liposomes comprised of 1-palmitoyl-2-oleoyl-*glycero*-3-phosphocholine (POPC) as a means to tune the release rate of compounds from liposomes.^6^ Mizukami *et al.* designed a protease-responsive membrane active peptide by modifying the AMP temporin L with a short branch containing a caspase-3 cleavable sequence.^12^ Proteolytic removal of the branch in the presence of liposomes loaded with fluorescent dyes resulted in release of the dye molecules. However, to function as a component of a DDS, the MAP should ideally be incorporated in or conjugated to the liposomes to achieve efficient co-delivery and triggered release at the target site.

To enable efficient co-delivery, MAPs can either be synthesized with lipid moieties inserted in liposomes during liposome formulation or be conjugated to liposomes with headgroup functionalized lipids. In addition to complicate peptide synthesis, the use of lipid-functionalized MAPs result in a liposomes with peptides on both the inner and the outer lipid leaflet.^13,14^ MAPs located on the inner leaflet could potentially interfere with drug loading and release are not available for interactions with proteases or other target enzymes that could be exploited for modulating the membrane activity of the MAPs. We have previously demonstrated the functionalization of liposome surfaces with *de novo* designed responsive and cysteine (Cys) containing MAPs to maleimide headgroup functionalized lipids (MPB-PE).^4,15–17^ MAP conjugation resulted in a peptide folding-dependent disruption of lipid membrane integrity. The membrane activity could be reversibly inhibited by introducing complementary peptides designed to heterodimerize with the MAPs.^4,17^ The effect was reversed upon proteolytic degradation of the complementary peptide, resulting in a protease triggered liposome content release.^4^ However, the development of protease responsive MAPs for triggered liposomal drug delivery is complicated by the strong tendency of cysteinyl thiols to oxidize to disulfides in aqueous buffers. Oxidized peptides will not react with Michael acceptors such as maleimides, which result in poor yield in the liposome conjugation step. From this perspective, strain-promoted azide–alkyne cycloaddition (SPAAC) has emerged as an interesting alternative for peptide–lipid conjugation. SPAAC reactions allow for the attachment of functional molecules to liposomes without the need for toxic catalysts, such as copper, making it particularly suitable for applications in drug delivery, imaging, and tissue engineering.^18^ SPAAC could potentially also enable an orthogonal method to Cys–maleimide based conjugation for functionalization of liposomes with two or more different peptides. Dibenzocyclooctyne (DBCO) is widely used for SPAAC and DBCO functionalized dox-containing liposomes have been explored in combination with a pre-targeting strategy based on azide-modified peptides for treatment of triple negative breast cancer.^19^ Moreover, coupling of DBCO modified liposomes to azide-labeled T-cells has been investigated for cell-mediated drug delivery.^20^ Conjugation of azide-modified AMPs^21^ or recombinant membrane proteins^22,23^ to DBCO functionalized liposomes have been explored for temporary inactivation of the AMP for safer delivery and for studies of membrane proteins in membrane-mimetic environments, respectively. However, the impact of the hydrophobic DBCO moiety on the properties of the lipid bilayer and the interaction with membrane active peptides have not been extensively studied.

**Scheme 1:**
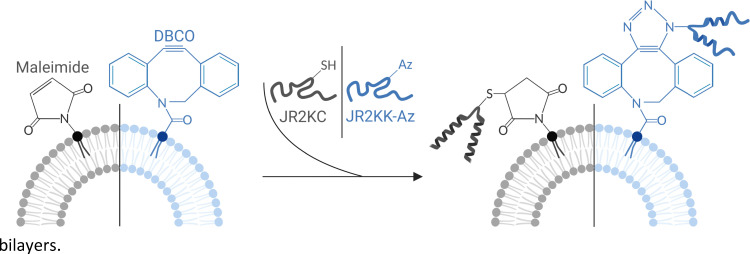
Illustration displaying the structures resulting from a Michael addition reaction between a maleimide functionalized lipid and a thiol-containing JR2KC peptide and a SPAAC reaction between a DBCO functionalized lipid and an azide-containing JR2KK-Az peptide.

Here we investigate and compare two different click reactions based on maleimide–thiol Michael addition and SPAAC for conjugation of membrane active peptides to liposomes. Furthermore, we explore how the choice of conjugation strategy influences the membrane activity of the peptides, the peptide-mediated release mechanism, and the orthogonality of the two reactions. As a MAP, we utilized the well-characterized *de novo* designed helix-loop-helix polypeptide JR2KC. JR2KC is an amphipathic and lysine (Lys) rich 42-residue peptide with a single Cys residue in the loop region. The peptide is unfolded in solution at pH 7 but adopts a well-defined α-helical conformation when covalently conjugated to lipid membranes using thiol–maleimide coupling (Scheme 1).^4^ Conjugation results in a peptide-folding dependent disruption of lipid membrane integrity, which enables detailed studies of the effect of conjugation and lipid bilayer properties on membrane activity. To allow for conjugation of JR2KC to DBCO-functionalized liposomes, the Cys residue was exchanged for a Lys with an azide-modified side chain, affording the peptide JR2KK-Az (Scheme 1).

We observed dramatic differences in the interactions of the peptide with the liposomes depending on the conjugation strategy used and the lipid membrane composition. Whereas thiol Michael addition of JR2KC to maleimide headgroup functionalized lipids triggered a rapid and peptide concentration-dependent liposomal release, SPAAC resulted in a slower and less efficient release. Moreover, significant cross-reactivity was seen, where the Cys containing peptide JR2KC triggered extensive release from DBCO-functionalized liposomes with 30 mol% cholesterol. Thus, the two click reactions were not mutually orthogonal unless SPAAC conjugation using JR2KK-Az was carried out prior addition of JR2KC. The cross-reactivity was more pronounced in cholesterol containing liposomes. Our findings demonstrate that both the conjugation strategy and the lipid composition have a dramatic impact on the function of membrane active peptides and their interaction with lipid bilayers.

## Experimental

### General

All chemicals used were purchased from Sigma Aldrich (Sigma-Aldrich, Saint Louis, Missouri, USA) apart from the lipids that were acquired from Avanti Polar Lipids (Alabaster, USA).

### Peptide synthesis

All peptides were obtained using Fmoc based solid-phase peptide synthesis. JR2KC (NAADLEKAIEALEKHLEAKGPCDAAQLEKQLEQAFEAFERAG) and JR2KCref (NaaDLEKaIEaLEKHLEaKGPCDaaQLEKQLEQaFEaFERaG) were synthesized as described earlier.^15^ JR2KK-Az (NAADLEKAIEALEKHLEAKGPK(Az)DAAQLEKQLEQAFEAFERAG) and JR2K (NAADLEKAIEALEKHLEAKGPVDAAQLEKQLEQAFEAFERAG) were synthesized on a Liberty Blue Automated Microwave Peptide Synthesizer (CEM, Matthews, North Carolina). The Lys-22 in JR2KK-Az was side-chain protected using an allyloxycarbonyl (Alloc) to allow site-selective modifications. Fmoc-Gly-Wang (Iris biotech GmbH) was used as solid support, and all couplings were performed twice using a 5-fold excess of amino acid. Base and coupling reagents were Oxyma pure and *N*,*N*′-diisopropylcarbodiimide (DIC), respectively, and microwave conditions was used throughout. Alloc-deprotection and subsequent azide functionalization was performed by hand outside of the synthesizer. The first was achieved by incubating the resins in 15 mL of trichloromethane/acetic acid/*N*-methylmorpholine (17 : 2 : 1), containing 3 equiv. of tetrakis(triphenylphosphine)palladium(0) (Pd(PPh_3_)_4_), for 3 h in a N_2_ atmosphere. Washing was sequentially performed with 20 mM diethyldithiocarbamic acid in DMF and 30 mM *N*,*N*-diisopropylethylamine in DMF, followed by DMF and DCM. The latter was performed by incubating the resins for 18 h in 3-azidopropionic acid (1 mmol), *N*-hydroxy-succinimide (1 mmol), *N*-ethyl-*N*′-(3-(dimethylamino)propyl) carbodiimide hydrochloride (1 mmol) and DIPEA (2.5 mmol) in DCM (10 mL). The crude peptides were cleaved from the solid supports, purified and verified as previously described (Fig. S1–S8, ESI†).^24^

### LUV preparation

Thin-film hydration followed by extrusion was used to create large unilamellar liposomes (LUVs). The lipid film was formed by mixing desired molar ratios of lipids dissolved in chloroform and then evaporating the solvent using a stream of nitrogen. The lipid film was placed in a desiccator overnight to ensure complete solvent removal. Liposomes were formed by rehydrating the lipid film with buffer for 10 min and subsequently vortex for 1 min, generating 5 mg ml^−1^ of total lipid solution. Monodisperse LUVs were achieved by extruding the lipid suspensions 21 times through a 100 nm polycarbonate membrane using a mini extruder (Avanti Polar Lipids, Alabaster, Alabama). Buffers used for rehydration included 0.01 M PBS (140 mM sodium chloride, 2.7 mM potassium chloride and 10 mM phosphate, pH 7.4) for DLS and FRET measurements and CF release experiments, 0.01 M phosphate (PB) pH 7.4 for measurements of CD and zeta potential and 50 mM CF (self-quenching concentrations) dissolved in 10 mM PB pH 7.4 with 90 mM NaCl for CF release experiments using fluorescence. For CF-loaded liposomes, unencapsulated CF was removed prior analysis by size exclusion chromatography using a G-25 column (Cytiva) and eluating with PBS.

### Carboxyfluorescein (CF) release assay

Peptide induced CF release from CF-loaded liposomes (self-quenching concentrations, 50 mM) over time was studied using a fluorescence plate reader (Tecan Infinite M1000 Pro, Tecan Austria GmbH, Grödig/Salzburg, Austria), *λ*_ex_ = 485 nm and *λ*_em_ = 520 nm. The lipid suspensions were diluted to a final lipid concentration of 40 μM using PBS (0.01 M, pH 7.4) in 96-well plates. For the sequential release assay, 20 μM of each liposome type was used giving a total concentration of CF encapsulating liposomes of 40 μM. Prior to peptide addition the background fluorescence (*F*_0_) was measured. Peptide was then added to a final volume of 200 μl and the fluorescence was measured over time (*F*). To achieve full release, the liposomes were incubated for 10 min with Triton X-100 (1% v/v) before the fluorescence was measured again (*F*_tot_). *F*_0_ and *F*_tot_ were measured three times for each well and the mean was used for calculations. CF-release at individual time points were then calculated using 
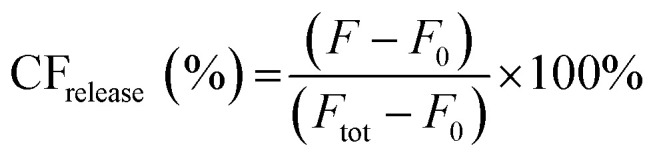
.

### Fluorescence resonance energy transfer (FRET)

0.5 mol% of each of the FRET pair 1,2-dipalmitoyl-*sn-glycero*-3-phosphoethanolamine-*N*-(7-nitro-2-1,3-benzoxadiazol-4-yl) (16 : 0 NBD, donor) and 1,2-dioleoyl-*sn-glycero*-3-phosphoethanolamine-*N*-(lissamine rhodamine B sulfonyl) (18 : 1 Rhod, acceptor) were included in the liposomes generating the four lipid compositions: 94 : 5 : 0.5 : 0.5 POPC/MPB/NBD/Rhod, 94 : 5 : 0.5 : 0.5 POPC/DBCO/NBD/Rhod, 64 : 5 : 30 : 0.5 : 0.5 POPC/MPB/Chol/NBD/Rhod and 64 : 5 : 30 : 0.5 : 0.5 POPC/DBCO/Chol/NBD/Rhod. The FRET ratio was measured on a Fluoromax-4 spectrophotometer (Horiba Jobin Yvon Inc., United States) by exciting the NBD-fluorophore at 460 nm and studying the fluorescence emission at 535 and 583 nm.^25^ The fluorescence was first measured on solely liposomes (40 μM in PBS) after which peptides were added (4 μM) and left to incubate for 1 h followed by a new measurement of the fluorescence intensities. The samples were then incubated with 1% v/v Triton X-100 for 10 min before measuring the emission intensity to find the unquenched fluorescence for normalization. All samples were recorded in triplicate. The FRET ratio was calculated using 
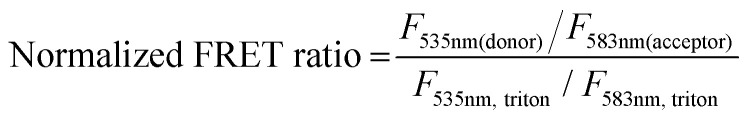
.

### Circular dichroism (CD) spectroscopy

CD measurements were recorded on a Chirascan (Applied Photophysics, Leatherhead, United Kingdom) scanning from 280 to 200 nm with steps of 0.5 nm, at room temperature using a 1 mm pathlength quartz cuvette. All samples were prepared in PB (0.01 M, pH 7.4), had a lipid and/or peptide concentration of 1.2 mM and 30 μM respectively, and incubated for 60 min before analyzed. Pure PB was used as background for peptide measurements while liposomes in PB were used as background for peptide–liposome measurements. The samples were recorded five times, except background references that were recorded three times, and averaged. The curves were then smoothed using Savitzky–Golat algorithm.

### Dynamic light scattering (DLS)

DLS measurements were carried out on a Malvern ZetaSizer Nano ZS90 (Malvern Panalytical, Malvern, Worcestershire, United Kingdom). The average of 30 cycles of 10 s runs was used to determine the hydrodynamic radius of liposomes, alone and with peptides. Peptide and liposomes concentrations were chosen to match CF release assay being 1 μM and 40 μM respectively. The PBS buffer (0.01 M, pH 7.4) was filtered through a 0.22 μm filter prior to usage. The results were fitted to the cumulant model using the ZetaSizer software.

### *ζ*-Potential

A Malvern ZetaSizer Nano ZS90 (Malvern Panalytical, Malvern, Worcestershire, United Kingdom) was used to measure the *ζ*-potential of liposomes prepared in 0.01 M PB (pH 7.4).

### Data fitting

All data from the total CF release experiments were fitted using a Hill equation 
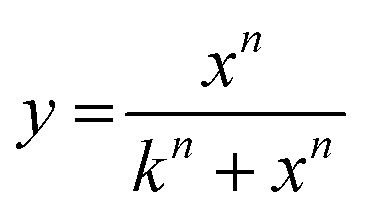
, where *n* = Hill coefficient, *x* = peptide concentration and *k* = constant.

### Surface plasmon resonance (SPR)

The SPR data were acquired using a Biacore T200 instrument and L1 sensor chips (Cytiva, Uppsala, Sweden). PBS (10 mM, pH = 7.4) was used as the running buffer for all experiments. The removal of negative charges in the dextran matrix was accomplished by injecting 250 μM EDC and 50 μM NHS for 600 seconds, followed by 1 mM ethanolamine for 210 seconds with a flow rate of 10 μL min^−1^, to reduce unspecific electrostatic binding to the sensor chip. The liposomes were injected at a 200 μM lipid concentration and a contact time of 900 seconds and a flow rate of 5 μL min^−1^, followed by running buffer subsequently flowed at 40 μL min^−1^ for 2 min. Peptides were injected at a concentration of 40 μM at a flow rate of 5 μL min^−1^ flow rate for 1200 seconds followed by 1200 seconds dissociation. Two successive 30 second injections of isopropanol : 50 mM NaOH, 2 : 3 at a flow rate of 10 μL min^−1^ were used for regeneration of the sensor chips. POPC liposomes were used as a reference in all experiments.

## Results and discussion

The two peptides JR2KC and JR2KK-Az (Table 1) were synthesized using solid phase peptide synthesis. Whereas JR2KC has a Cys residue in position 22, JR2KK-Az carries a Lys with an azide modified side chain in this position for conjugation to maleimide and DBCO headgroup modified lipids, respectively. The effect of conjugation of the two peptides to large unilamellar liposomes (LUVs) comprised of 1-palmitoyl-2-oleoyl-*glycero*-3-phosphocholine (POPC) and 1,2-dioleoyl-*sn-glycero*-3-phosphoethanolamine-*N*-[4-(*p*-maleimidophenyl)butyramide] (MPB-PE) or 1,2-dioleoyl-*sn-glycero*-3-phosphoethanolamine-*N*-dibenzocyclooctyne (DBCO-PE), with and without 30 mol% cholesterol (Chol), was explored. The liposomes were prepared using the thin-film hydration method followed by extrusion through a polycarbonate membrane with 100 nm pores. The conjugation specificity of JR2KC and JR2KK-Az to liposomes with 5 mol% of either MPB-PE (MPB liposomes) or DBCO-PE (DBCO liposomes) without Chol or with 30% Chol (MPB/Chol and DBCO/Chol liposomes) was assessed by monitoring the conjugation-dependent release of carboxyfluorescein (CF).

**Table 1:** Primary sequence of JR2KC, JR2KK-Az, JR2K, and JR2KC_ref_

JR2KC	NAADLEKAIEALEKHLEAKGP**C**DAAQLEKQLEQAFEAFERAG
JR2KK-Az	NAADLEKAIEALEKHLEAKGP**K(Az)**DAAQLEKQLEQAFEAFERAG
JR2K	NAADLEKAIEALEKHLEAKGPVDAAQLEKQLEQAFEAFERAG
JR2KC_ref_	NaaDLEKaIEaLEKHLEaKGP**C**DaaQLEKQLEQaFEaFERaG

**Fig. 1:**
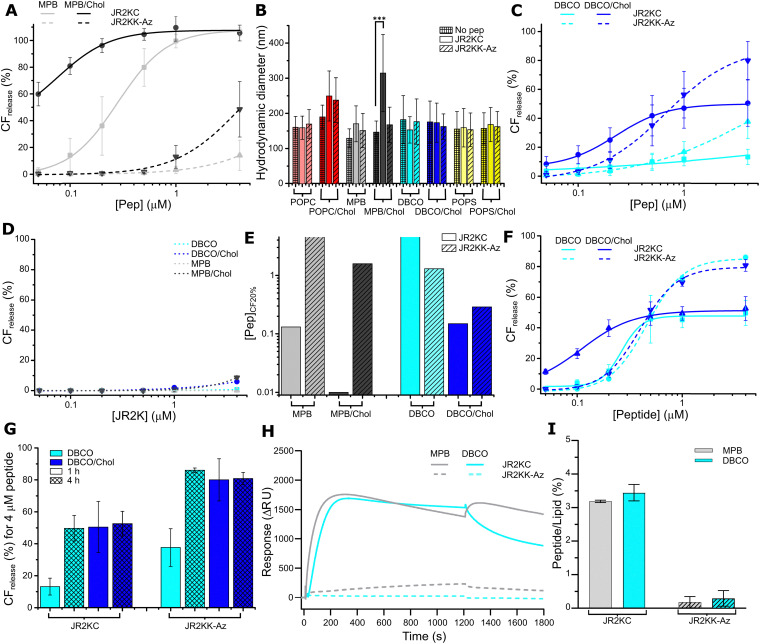
(A) Total CF release after 1 h incubation with JR2KC (solid) and JR2KK-Az (dashed) on 40 μM MPB liposomes, with (black) or without (grey) cholesterol. Data were fitted to a Hill equation, *N* ≥ 8. (B) Hydrodynamic diameter of liposomes (40 μM) alone and after incubation with 1 μM JR2KC or JR2KK-Az for 1 h. (*p*-value: * < 0.05, ** < 0.005 and *** < 0.0005). (C) Total CF release after 1 h incubation with JR2KC (solid) and JR2KK-Az (dashed) on 40 μM DBCO liposomes, with (blue) or without (cyan) cholesterol. Data were fitted to a Hill equation, *N* ≥ 8. (D) Total CF release from 40 μM MPB (grey) and MPB/Chol liposomes (black), and DBCO (cyan) and DBCO/Chol liposomes (blue), after 1 h incubation with JR2K. Data were fitted to a Hill equation, *N* ≥ 3. (E) Peptide concentration required to reach 20% CF release after 1 h incubation ([Pep]_CF20%_) estimated from the fittings in (A) and (C). (F) Total CF release after 4 h incubation with JR2KC (solid) and JR2KK-Az (dashed) on liposomes (40 μM) with DBCO, with (blue) or without (cyan) cholesterol. Data were fitted to a Hill equation, *N* = 3. (G) Total CF release from DBCO-containing liposomes, with (blue) or without (cyan) cholesterol, obtained after 1 and 4 h, respectively, incubation with 4 μM JR2KC or JR2KK-Az. Data obtained from (C) and (F). (H) SPR sensorgrams showing the interaction of JR2KC and JR2KK-Az with MPB and DBCO liposomes on a Biacore L1 sensor chip. (I) Number of peptides per lipid after 10 min injection of JR2KC and JR2KK-Az over a MPB and DBCO liposome functionalized sensor chip.

In the absence of peptides, no CF release could be observed for any of the lipid compositions used, neither with nor without cholesterol (Fig. S9, ESI†). Additionally, no CF release was observed from liposomes lacking MPB and DBCO after addition of JR2KC or JR2KK-Az, neither with nor without cholesterol (Fig. S10, ESI†). However, addition of JR2KC to MPB liposomes caused rapid CF release for concentrations of JR2KC ≥ 0.1 μM (Fig. 1(A) and Fig. S11A, ESI†), which is in agreement with previous work.^4,15^ No significant changes in hydrodynamic diameter (*D*_H_) were seen after addition of JR2KC to MPB liposomes indicating that conjugation did not result in aggregation or dissolution of the liposomes (Fig. 1(B) and Fig. S12A, ESI†). Moreover, on par with previous observations,^15^ JR2KC triggered more extensive and faster CF release from MPB/Chol liposomes (Fig. 1(A) and Fig. S11B, ESI†) and a lower peptide concentration (0.05 μM) was required to initiate CF release in MPB/Chol liposomes compared to MPB liposomes (0.1 μM). We have previously confirmed that including cholesterol results in a peptide-mediated lipid phase separation where peptides co-localize in large domains, which promoted rapid and peptide folding-dependent CF release.^15^ In contrast to MPB liposomes, addition of JR2KC to MPB/Chol liposomes triggered a significant increase in *D*_H_ of the liposomes (Fig. 1(B) and Fig. S12B, ESI†), which could be an effect of the accumulation of high concentrations of peptides on the liposome surface or swelling of the liposomes due to peptide insertion in the lipid bilayer. Minor aggregation of the liposomes can, however, not be excluded.

As expected, addition of JR2KK-Az to DBCO liposomes was found to trigger CF release (Fig. 1(C) and Fig. S13A, ESI†). However, compared to the corresponding JR2KC mediated release from MPB liposomes, the CF release was significantly slower. Higher release rates and more extensive release were seen for DBCO/Chol liposomes (Fig. 1(C) and Fig. S13B, ESI†), but still slower release than triggered by JR2KC from MPB liposomes. After 1 hour incubation, JR2KK-Az caused about 80% CF release from DBCO/Chol liposomes, compared to 100% CF release in 10 min for JR2KC interacting with MPB/Chol liposomes. There were no substantial differences in zeta potential between DBCO and MPB liposomes (Fig. S6, ESI†) or between DBCO/Chol and MPB/Chol liposomes. However, both DBCO/Chol and MPB/Chol liposomes showed a more negative zeta potential than the corresponding non-cholesterol containing liposomes. Increasing the ratio of cholesterol in POPC-liposomes has been reported to reduce Na^+^ binding to lipid headgroups, resulting in a more negative zeta potential.^26^ The lower zeta potential of the cholesterol containing liposomes could result in a more extensive accumulation of peptides at the lipid bilayer compared to MPB and DBCO liposomes without cholesterol. However, the observed difference in release kinetics between JR2KC and JR2KK-Az interacting with MPB/Chol and DBCO/Chol liposomes, respectively, cannot be due to differences in electrostatically driven preconcentration effects but rather a consequence of the differences in the rate of the two different click reactions. The thiol–maleimide reaction is rapid compared to most SPAAC reactions with reported second-order rate constant above 700 M^−1^ s^−1^.^27^ SPAAC ligations based on DBCO derivatives typically display second-order rate constants in the range of 1–2 M^−1^ s^−1^ when reacting with azide groups.^28^ Moreover, the DBCO group is significantly more hydrophobic than the maleimide moiety and could likely interact with, and reside inside, the hydrophobic lipid bilayer, restricting its availability to react with the peptides, which could further contribute to the slower release. In contrast to JR2KC interacting with MPB/Chol, no changes in *D*_H_ were seen after addition of JR2KK-Az to DBCO and DBCO/Chol liposomes (Fig. 1(B) and Fig. S12C, D, ESI†), which indicate a different mode of peptide–lipid interaction or accumulation of fewer peptides at the liposome surface.

To investigate the orthogonality of the two different click reactions we exposed MPB liposomes to JR2KK-Az and DBCO liposomes to JR2KC. As expected, JR2KK-Az did not cause any substantial CF release from MPB liposomes (Fig. 1(A) and Fig. S15A, ESI†). However, in MPB/Chol liposomes, the highest JR2KK-Az concentration tested (4 μM), caused about 45% CF release after 1 h incubation (Fig. 1(A) and Fig. S15B, ESI†). To exclude unspecific electrostatic interactions as the cause for observed CF release, we prepared liposomes with 5 mol% POPS, *i.e.*, POPS (POPC : POPS 95 : 5) and POPS/Chol (POPC : POPS : Chol 65 : 5 : 30) liposomes. The zeta potential of POPS and POPS/Chol liposomes were in the same range as the corresponding MPB liposomes (Fig. S14, ESI†). However, no release was seen for any of the POPS liposomes (Fig. S16, ESI†), which indicates that the CF release caused by JR2KK-Az from MPB/Chol liposomes was indeed triggered by conjugation-dependent interactions. This was further confirmed using a peptide (JR2K) with a valine in position 22 instead of Cys or Lys-Az (Table 1). JR2K can not react with neither maleimides nor DBCO groups and did not trigger any release from any of the liposome compositions (Fig. 1(D)). Maleimides and azides do not typically react directly with each other under standard conditions but [3+2] dipolar cycloaddition between azide- and maleimide-containing molecules has been reported when the reacting molecules were favorably preorganized by including recognition sites to the reactive partners.^29^ The electrostatically driven accumulation of JR2KK-Az on the MPB/Chol liposomes hence likely resulted in a preorganization effect that positioned the peptide azide group in close vicinity of the maleimide moiety in MPB-PE allowing for the corresponding triazoline cycloadducts to form. The orthogonality of the two different click reactions explored here was consequently found to be dependent on the cholesterol content in the lipid bilayer, which to our knowledge has not previously been reported. However, since no significant increase in *D*_H_ was seen after addition of JR2KK-Az to MPB or MPB/Chol liposomes (Fig. 1(B) and Fig. S12A, B, ESI†), the resulting peptide surface concentration was likely lower than for JR2KC on MPB/Chol liposomes at the same concentrations or resulted in a different organization of the peptides at the liposome surface, which correlates with the observed lower CF release efficiency.

When incubating JR2KC with DBCO liposomes, a small amount of CF release was seen, corresponding to about 13% for the highest JR2KC concentration (Fig. 1(C) and Fig. S17A, ESI†). In contrast, addition of JR2KC to DBCO/Chol liposomes, resulted in rapid CF release also for the lowest peptide concentrations tested (Fig. 1(C) and Fig. S17B, ESI†). The concentration of JR2KC required to trigger 20% CF release ([Pep]_CF20%_) from DBCO/Chol liposomes during 1 h incubation was even lower than for JR2KK-Az (Fig. 1(E) and Table 2), which is surprising. Also, the lowest concentration of JR2KC tested (0.05 μM) caused about 10% CF release. However, whereas JR2KK-Az eventually caused almost complete CF release, the JR2KC triggered release leveled out around 50%. Increasing the incubation time to 4 h did not result in any additional CF release (Fig. 1(F) and (G)). Moreover, maximum release was seen already for JR2KC concentrations of around 0.5 μM and increasing the concentration further did not lead to more extensive CF release. No CF release was seen when exposing the DBCO/Chol liposomes to JR2K, confirming that the release was conjugation dependent (Fig. 1(D)). Azide-independent reactions with alkynes has been extensively reported when using SPAAC for protein and peptide labeling, which likely is a result of a thiol–yne reaction with Cys containing biomolecules.^30–32^ Addition of JR2KC to DBCO/Chol liposomes did not influence the colloidal stability of the liposome compositions and did not result in any significant changes in *D*_H_ (Fig. 1(B) and Fig. S12D, ESI†). The JR2KC triggered release from DBCO/Chol liposomes consequently displayed distinctly different characteristics compared to when interacting with MPB/Chol liposomes. The CF release caused by JR2KC from DBCO and DBCO/Chol liposomes could thus be a result of both a preorganization effect and an enhancement of the thiol–yne reaction rate induced by the local microenvironment in the lipid bilayer.

**Table 2:** Peptide concentrations required to achieve 20% CF release after 1 h incubation ([Pep]_CF20%_) on the different liposome compositions

[Pep]_CF20%_ (μM)	JR2KC	JR2KK-Az	JR2KC_ref_	JR2K
MPB	0.13	—	—	—
MPB/Chol	<0.05	1.57	2.16	—
DBCO	—	1.30	—	—
DBCO/Chol	0.15	0.29	3.06	—

Blenke *et al.* has reported that liposomes with DBCO functionalized lipids with a 2 kDa poly(ethylene glycol) spacer showed tendencies to aggregate when incorporated at higher ratios than 2% because of the burial of the hydrophobic DBCO group in the lipid bilayer.^21^ No such tendencies were observed here for the DBCO liposomes as confirmed by dynamic light scattering (DLS) (Fig. 1(B) and Fig. S12C, D, ESI†), likely due to the shorter spacer between the lipid headgroup and the DBCO moiety of the DBCO-PE lipid used in this work, which limits the possibilities for the DBCO group to interact with the bilayer of other liposomes. However, the DBCO group may still interact with the lipid bilayer of the individual liposomes, which will influence both accessibility, reactivity, and lipid membrane properties. Despite being apparently less accessible, the DBCO group is most likely reactive also when buried in the lipid bilayer. In fact, the SPAAC reaction between a membrane protein labeled with an azide group in the membrane spanning region and a DBCO group coupled to a hydrophilic Alexa dye has been reported to increase in efficiency by 3-orders of magnitude within the hydrophobic core of micelles.^33^ Bilayer confined SPAAC has also recently been demonstrated using a liposome fusion process to bring lipophilic dyes functionalized with SPAAC reactive handles into close contact.^34^ Thus, the hydrophobic environment in the lipid bilayer could potentially also increase the rate of the thiol–yne reaction between JR2KC and the DBCO group contributing to the rapid CF release observed from the DBCO/Chol liposomes. The peptide concentrations required to achieve 20% CF release after 1 h incubation ([Peptide]_CF20%_) on the different liposome compositions for all the different peptides explored are summarized in Table 2.

Surface plasmon resonance (SPR) was used to further investigate the role of the anchoring moiety on the conjugation process. Liposomes (100 μM) containing either 5 mol% MPB-PE or 5 mol% DBCO were first immobilized on a Biacore L1 chip. Peptides (40 μM) were then injected and the SPR response was recorded. Liposomes lacking MPB-PE or DBCO (100% POPC) were used as negative control and for background subtraction. The SPR responses showed a rapid and substantial binding of JR2KC to MPB liposomes with no apparent dissociation (Fig. 1(H)), indicating efficient thiol–ene conjugation (Fig. 1(A)). The slight negative base-line shift could indicate some peptide-conjugation induced loss of lipids. About 60% of the available maleimide groups in the immobilized MPB liposomes were consumed after a 10 min injection of JR2KC (Fig. 1(I)), which is consistent with the observed CF release. Furthermore, the SPR responses show binding of the JR2KK-Az peptide to DBCO liposomes, although with significantly slower kinetics and a lower response compared to JR2KC binding to MPB liposomes (Fig. 1(H) and (I)). After 20 minutes, the peptide-to-lipid ratio for JR2KK-Az bound to DBCO liposomes is approximately sixfold lower than that of JR2KC to MPB liposomes. This slower binding correlates with the slower CF release kinetics observed for JR2KK-Az on DBCO liposomes (Fig. 1(C)), suggesting that the fraction of JR2KK-Az bound to DBCO liposomes may gradually converge with that of JR2KC to MPB liposomes over time. The SPR data further confirmed that JR2KC accumulated extensively on DBCO liposomes whereas JR2KK-Az showed limited binding to MPB liposomes, which is in line with the observed CF release (Fig. 1(A) and (C)). The interaction of JR2KC with DBCO liposome, however, appeared to be relatively dynamic resulting in substantial dissociation of peptides after the injection.

### Folding partitioning coupling

**Fig. 2:**
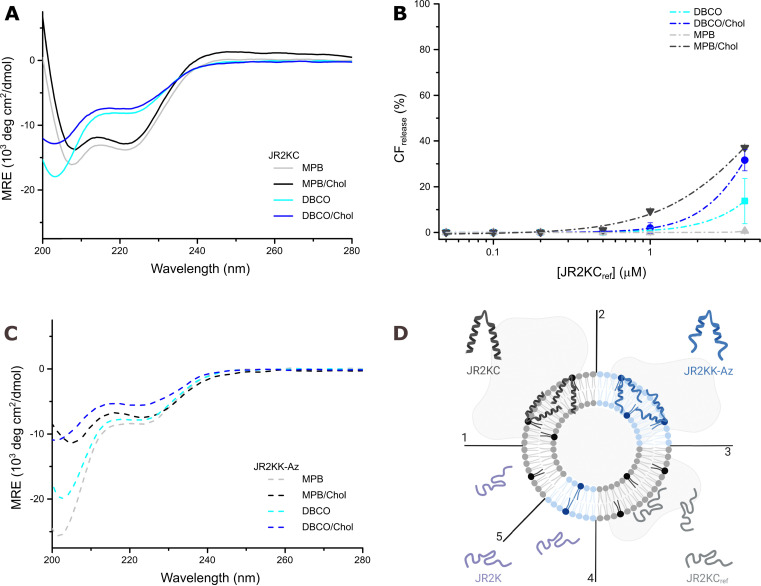
(A) CD spectra of 30 μM JR2KC incubated for 1 h with 1.2 mM MPB (grey), MPB/Chol (black), DBCO (cyan), and DBCO/Chol (blue) liposomes. (B) Total CF release from 40 μM MPB (grey), MPB/Chol (black), DBCO (cyan), and DBCO/Chol (blue) liposomes, after 1 h incubation with JR2KC_ref_. Data were fitted to a Hill equation, *N* ≥ 3. (C) CD spectra of 30 μM JR2KK-Az incubated for 1 h with 1.2 mM MPB (grey), MPB/Chol (black), DBCO (cyan), and DBCO/Chol (blue) liposomes. (D) Illustration showing the lipid interactions of the different peptide–liposome combinations: (1) JR2KC bind rapidly to MPB liposomes, resulting in folding and rapid liposome cargo release; (2) JR2KK-Az bind to DBCO liposomes, resulting in partial folding and slow release; (3) JR2KC_ref_ associate dynamically to MPB liposomes, remain unstructured and cause limited release; (4) JR2K interacting with DBCO liposomes, and; (5) JR2K interacting with MPB liposomes, result in no folding and no release.

JR2KC and JR2KK-Az are amphipathic and have a distinct hydrophobic face when folded into an α-helix, but their high charge density (+11 at pH 7) will constrain the organization of the peptide on the liposomes surface and most likely prevent full insertion into the hydrophobic core of the lipid bilayer, which probably influences how the peptides organize and fold during and after conjugation. Both JR2KC and JR2KK-Az are random-coil in solution at pH 7 (Fig. S18A and B, ESI†). Conjugation of JR2KC to MPB liposomes resulted in folding of the peptide into a well-defined α-helix both in the presence and absence of cholesterol (Fig. 2(A) and Table 3), in line with previous results.^4,15^ When exposed to liposomes without MPB-PE, JR2KC remained as a random coil (Fig. S18A, ESI†). To further investigate the role of folding and conjugation on lipid membrane destabilization and CF release for the different peptide–liposome combinations a fourth peptide (JR2KC_ref_) was introduced (Table 1). JR2KC_ref_ has the identical amino acid sequence as JR2KC but is unable to fold due to isomer exchange of all l-alanine residues (l-Ala) by d-alanine (d-Ala). No CF release was seen upon the addition of JR2KC_ref_ to MPB liposomes (Fig. 2(B)).

**Table 3:** MRE_222/208_ ratio for JR2KC and JR2KK-Az after 1 h incubation with MPB and DBCO liposomes, with and without 30% cholesterol

	MPB	MPB/Chol	DBCO	DBCO/Chol
JR2KC	0.85	0.91	0.58	0.75
JR2KK-Az	0.47	0.70	0.52	0.78

In addition, SPR revealed limited binding of JR2KC_ref_ to MPB liposomes (Fig. S19, ESI†), which clearly indicates that folding of JR2KC promoted the accumulation of the peptides at the lipid bilayer. However, despite being unable to fold and bind to the liposomes, JR2KC_ref_ triggered certain CF release from MPB/Chol liposomes at concentrations above 1 μM, but significantly less compared to JR2KC (Fig. 2(B)). We can thus conclude that the CF release caused by JR2KC in MPB liposomes is highly folding-dependent. However, in MPB/Chol liposomes, non-folding dependent interactions caused by the dynamic interactions of the cationic peptides at the lipid membrane could contribute to lipid membrane destabilization. Addition of JR2KK-Az to MPB/Chol liposomes resulted in a change in secondary structure indicating that the observed CF release might be an effect of folding-partition coupling (Fig. 2(C) and Table 3). The tendencies for folding in JR2KK-Az also aligns with previous results showing that having a Lys-Az instead of a Cys in the loop region of a similar helix-loop-helix peptides does not affect the ability of the peptide to fold.^35,36^ Interestingly, JR2KK-Az showed no folding when incubated with DBCO or DBCO/Chol liposomes (Fig. 2(C) and Table 3), despite triggering substantial CF release (Fig. 1(C)). However, it should be noted that the slow binding of JR2KK-Az to DBCO liposomes indicated in the SPR response (Fig. 1(H) and (I)) could contribute to a less apparent folding in the CD spectra due to the low fraction of conjugated peptide present in the sample. The same trends, with no changes in secondary structure, were also seen for JR2KC interacting with DBCO and DBCO/Chol liposomes (Fig. 2(A) and Table 3). Yet, the CF release triggered by JR2KC_ref_ from DBCO and DBCO/Chol liposomes was much less pronounced (Fig. 2(B)), which indicates that the ability to fold still contributed to the peptide–lipid membrane interactions for the DBCO-containing liposomes. Due to the absence of defined secondary structure in the CD spectrum, any folding-dependent interactions between JR2KC and DBCO/Chol liposomes must be transient and very dynamic. The interpretation of the peptide–lipid interactions for some of the different peptide and liposome combinations are summarized in Fig. 2(D).

### Lipid phase separation

**Fig. 3:**
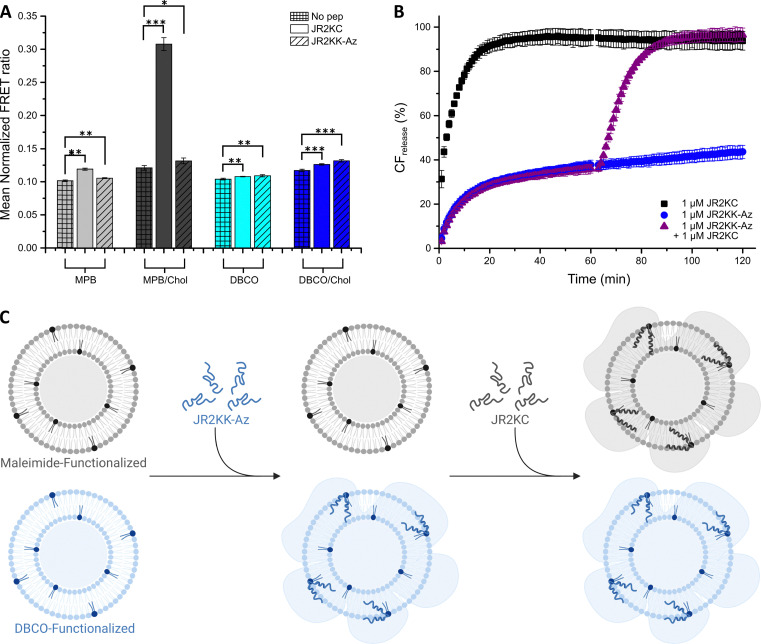
(A) Mean normalized FRET ratio of 40 μM MPB liposomes (black) and DBCO liposomes (blue), with (dark color) or without (light color) cholesterol, alone (meshed) or after 1 h incubation with 4 μM JR2KC (clear) and JR2KK-Az (diagonals), respectively. (*p*-value: * < 0.05, ** < 0.005 and *** < 0.0005) (B) CF release kinetics using both MPB and DBCO/Chol liposomes (20 μM of each, ratio 1 : 1) incubated with 1 μM JR2KC (black) or 1 μM JR2KK-Az (blue) during 120 min or with a sequential addition where 1 μM JR2KC was added after the initial first 60 min incubation with 1 μM JR2KK-Az (purple). (C) Schematic illustration of the selective sequential release experiment in (B). A sample with equal concentrations of MPB (grey) and DBCO/Chol (blue) liposomes was first exposed to JR2KK-Az resulting in JR2KK-Az triggered release from the DBCO liposomes only. In the second step, addition of JR2KC resulted in JR2KC-triggered release from the MPB liposomes.

We have previously demonstrated that the conjugation of JR2KC to MPB/Chol liposomes resulted in a lipid phase separation and formation of peptide rich domains, possibly facilitated by electrostatic interactions between the positively charged peptide and the negatively charged headgroup-functionalized lipids.^15^ To investigate if the difference in the interactions between JR2KC and JR2KK-Az and the different liposomes were influenced by differences in lipid phase behavior, we included 0.5 mol% of each of the fluorescence resonance energy transfer (FRET) pair 1,2-dipalmitoyl-*sn-glycero*-3-phosphoethanolamine-*N*-(7-nitro-2-1,3-benzoxadiazol-4-yl) (NBD, donor) and 1,2-dioleoyl-*sn-glycero*-3-phosphoethanolamine-*N*-(lissamine rhodamine B sulfonyl) (Rhod, acceptor) in the liposomes, generating the four lipid compositions: POPC : MPB : NBD : Rhod (94 : 5 : 0.5 : 0.5), POPC : DBCO : NBD : Rhod (94 : 5 : 0.5 : 0.5), POPC : MPB : Chol : NBD : Rhod, (64 : 5 : 30 : 0.5 : 0.5), and POPC : DBCO : Chol : NBD : Rhod (64 : 5 : 30 : 0.5 : 0.5). The increase in the FRET ratio after addition of JR2KC and JR2KK-Az to MPB or DBCO liposomes, respectively, was very small which was expected since cholesterol is typically a requirement for formation of the liquid ordered (l_o_) phase (Fig. 3(A)). In contrast, a clear increase in the FRET ratio was seen after the addition of JR2KC to MPB/Chol liposomes, from 0.12 to 0.31, supporting our previous observations that conjugation of JR2KC triggers a lipid phase separation in cholesterol rich liposomes. This process is likely driven by interactions between the conjugated peptides, facilitated by the change in lipid packing and fluidity mediated by cholesterol. The increase in the FRET ratio upon addition of JR2KK-Az to MPB/Chol liposomes or DBCO/Chol liposomes was significantly smaller and comparable to the values observed for the corresponding liposomes without cholesterol. Likewise, no clear phase separation was induced by JR2KC interacting with DBCO or DBCO/Chol liposomes. The lateral interactions between peptides conjugated to the DBCO/Chol liposomes were thus likely less pronounced compared to JR2KC conjugated to MPB/Chol liposomes, further confirming different conjugation-dependent modes of interactions between the peptides and the lipid bilayer.

### Sequential release

To further explore the orthogonality of the two conjugation strategies, we explored possibilities to sequentially and specifically target MPB and DBCO liposomes using the different peptides in samples containing both types of liposomes. In a suspension containing equal concentrations of MPB liposomes and DBCO/Chol liposomes, addition of 1 μM JR2KC triggered rapid and full release from all liposomes due to the cross-reactivity of the peptide (Fig. 3(B)). In contrast, addition of 1 μM JR2KK-Az only caused about 45% CF release, corresponding to almost full release from about half of the liposome population, which is consistent with the absence of cross-reactivity between JR2KK-Az and MPB liposomes at this peptide concentration (Fig. 1(A)). The orthogonality of the reaction was confirmed by loading the DBCO/Chol liposomes with PBS buffer instead of CF. When combined with CF loaded MPB liposomes, addition of JR2KK-Az did not result in any CF release, clearly demonstrating the absence of cross-reactivity (Fig. S20, ESI†). However, in a sample containing equal concentrations of CF loaded MPB liposomes and DBCO/Chol liposomes, the addition of JR2KC after addition of JR2KK-Az resulted in full and sequential CF release from both types of liposomes, showing the possibility to target and trigger release from the two different types of liposomes with high selectivity (Fig. 3(C)).

## Conclusions

In conclusion, we show that membrane active peptides can be conjugated to liposomes using both SPAAC and thiol–maleimide Michael addition reactions, resulting in perturbations in lipid membrane integrity. The two conjugation strategies were under certain circumstances orthogonal but the accumulation of the peptides on cholesterol rich liposomes (MPB/Chol and DBCO/Chol) was found to contribute to both non-azide dependent thiol-yne reactions between the cysteine thiol group of JR2KC and the DBCO moiety, as well as a [3+2] dipolar cycloaddition between the azide group in JR2KK-Az and the maleimide (MPB) group. To our knowledge, the impact of lipid membrane fluidity and organization caused by addition of cholesterol on click reactions has not previously been reported. Moreover, whereas JR2KC triggered a conjugation-dependent CF release from both MPB and MPB/Chol liposomes that was folding-dependent and resulted in folding of the peptide, the conjugation of JR2KC and JR2KK-Az to DBCO and DBCO/Chol liposomes demonstrated a more complex behavior. The CF release was indeed both conjugation- and folding-dependent but no defined secondary structure was observed, indicating transient and very dynamic folding-dependent interactions between the peptides and the lipid bilayer. The conjugation of JR2KC to DBCO/Chol liposomes resulted in rapid but incomplete CF release whereas JR2KK-Az triggered a slower but almost complete CF release from DBCO and DBCO/Chol liposomes. SPR analysis confirmed efficient thiol–ene conjugation of JR2KC to MPB-PE liposomes and that JR2KC also accumulated at DBCO liposomes, while JR2KK-Az binding to DBCO liposomes was significantly slower. Moreover, JR2KC triggered a distinct lipid phase separation when conjugated to MPB/Chol liposomes, which was not seen in the DBCO/Chol liposomes. The organization of the hydrophobic DBCO group in the lipid bilayer likely contributes to the observed differences, both influencing the reactivity with the thiol group and the organization of the peptides at the lipid bilayer.

The findings presented here provide mechanistic insight relevant for the development of protease-responsive liposomal systems, in which MAPs are rendered inactive through transient interactions or modifications and subsequently activated by enzymatic cleavage. Our comparative analysis of thiol–maleimide and SPAAC conjugation strategies highlights how conjugation kinetics, lipid accessibility, and membrane composition influence peptide–liposome interactions and release behavior. These results underscore the importance of selecting appropriate conjugation chemistry based on the desired therapeutic context, with maleimide-based approaches offering rapid and efficient functionalization, while SPAAC may enable orthogonal or multi-step functionalization schemes. Together, this work advances the design of responsive liposomal platforms with tunable release profiles for targeted drug delivery applications.

## Author contributions

Conceptualization: Daniel Aili; methodology: Alexandra Iversen, Johanna Utterström, Basab Kanti Das, Lalit Pramod Khare; investigation: Alexandra Iversen, Johanna Utterström, Basab Kanti Das, Lalit Pramod Khare; formal analysis: Alexandra Iversen, Johanna Utterström, Basab Kanti Das; resources: Daniel Aili; writing – original draft: Alexandra Iversen, Johanna Utterström; writing – review & editing: Alexandra Iversen, Johanna Utterström, Basab Kanti Das, Lalit Pramod Khare, Daniel Aili; supervision: Daniel Aili; funding acquisition: Daniel Aili.

## Conflicts of interest

There are no conflicts to declare.

## Supplementary Material

TB-013-D5TB00304K-s001

## Data Availability

Data for this article, including CF release data, CD spectra, and DLS data, are available at Zenodo at 10.5281/zenodo.14830442.

## References

[cit1] Olusanya T. O. B., Ahmad R. R. H., Ibegbu D. M., Smith J. R., Elkordy A. A. (2018). Molecules.

[cit2] Sercombe L., Veerati T., Moheimani F., Wu S. Y., Sood A. K., Hua S. (2015). Front. Pharmacol..

[cit3] Torchilin V. P. (2014). Nat. Rev. Drug Discovery.

[cit4] Lim S. K., Sandén C., Selegård R., Liedberg B., Aili D. (2016). Sci. Rep..

[cit5] Dathe M., Schumann M., Wieprecht T., Winkler A., Beyermann M., Krause E., Matsuzaki K., Murase O., Bienert M. (1996). Biochemistry.

[cit6] Sun L., Hristova K., Wimley W. C. (2021). Nanoscale.

[cit7] Zhang Q., Ran R., Zhang L., Liu Y., Mei L., Zhang Z., Gao H., He Q. (2015). J. Controlled Release.

[cit8] Sani M. A., Separovic F. (2016). Acc. Chem. Res..

[cit9] Mahlapuu M., Håkansson J., Ringstad L., Björn C. (2016). Front. Cell. Infect. Microbiol..

[cit10] Mäe M., Myrberg H., El-Andaloussi S., Langel Ü. (2009). Int. J. Pept. Res. Ther..

[cit11] Zhang Y., Wang C., Zhang W., Li X. (2023). Biomater. Transl..

[cit12] Mizukami S., Kashibe M., Matsumoto K., Hori Y., Kikuchi K. (2017). Chem. Sci..

[cit13] Daudey G. A., Zope H. R., Voskuhl J., Kros A., Boyle A. L. (2017). Langmuir.

[cit14] Daudey G. A., Schwieger C., Rabe M., Kros A. (2019). Langmuir.

[cit15] Utterström J., Barriga H. M. G., Holme M. N., Selegård R., Stevens M. M., Aili D. (2022). Bioconjugate Chem..

[cit16] Skyttner C., Selegård R., Larsson J., Aronsson C., Enander K., Aili D. (2019). Biochim. Biophys. Acta, Biomembr..

[cit17] Skyttner C., Enander K., Aronsson C., Aili D. (2018). Langmuir.

[cit18] Yoon H. Y., Lee D., Lim D. K., Koo H., Kim K. (2022). Adv. Mater..

[cit19] Zhang C., Wang J., Chen H., Ouyang M., Li Y., Tan C., Jiang Y., Tan Y. (2024). Adv. Funct. Mater..

[cit20] Li X., Weller S., Clergeaud G., Andresen T. L. (2024). Biotechnol. J..

[cit21] Oude Blenke E., Sleszynska M., Evers M. J. W., Storm G., Martin N. I., Mastrobattista E. (2017). Bioconjugate
Chem..

[cit22] Zhang H., Weingart J., Jiang R., Peng J., Wu Q., Sun X. L. (2013). Bioconjugate Chem..

[cit23] Wang L., Jiang R., Liu Y., Cheng M., Wu Q., Sun X. L. (2017). J. Biosci. Bioeng..

[cit24] Iversen A., Utterström J., Selegård R., Aili D. (2024). ACS Omega.

[cit25] Vu T. Q., Sant’Anna L. E., Kamat N. P. (2023). Biomacromolecules.

[cit26] Magarkar A., Dhawan V., Kallinteri P., Viitala T., Elmowafy M., Róg T., Bunker A. (2014). Sci. Rep..

[cit27] Saito F., Noda H., Bode J. W. (2015). ACS Chem. Biol..

[cit28] Gordon C. G., MacKey J. L., Jewett J. C., Sletten E. M., Houk K. N., Bertozzi C. R. (2012). J. Am. Chem. Soc..

[cit29] Sinclair A. J., Del Amo V., Philp D. (2009). Org. Biomol. Chem..

[cit30] Van Geel R., Pruijn G. J. M., Van Delft F. L., Boelens W. C. (2012). Bioconjugate Chem..

[cit31] Zhang C., Dai P., Vinogradov A. A., Gates Z. P., Pentelute B. L. (2018). Angew. Chem., Int. Ed..

[cit32] Ekkebus R., Van Kasteren S. I., Kulathu Y., Scholten A., Berlin I., Geurink P. P., De Jong A., Goerdayal S., Neefjes J., Heck A. J. R., Komander D., Ovaa H. (2013). J. Am. Chem. Soc..

[cit33] Tian H., Sakmar T. P., Huber T. (2015). ChemBioChem.

[cit34] Jumeaux C., Spicer C. D., Charchar P., Howes P. D., Holme M. N., Ma L., Rose N. C., Nabarro J., Fascione M. A., Rashid M. H., Yarovsky I., Stevens M. M. (2024). Angew. Chem., Int. Ed..

[cit35] Selegård R., Aronsson C., Brommesson C., Dånmark S., Aili D. (2017). Sci. Rep..

[cit36] Aronsson C., Jury M., Naeimipour S., Boroojeni F. R., Christoffersson J., Lifwergren P., Mandenius C.-F., Selegård R., Aili D. (2020). Biofabrication.

