# Supplementary material for: Orthogonal conjugation of anchoring-dependent membrane active peptides for tuning of liposome permeability
Source: J Mater Chem B. 2025 Jul 10;13(33):10267–77. doi: 10.1039/d5tb00304k (PMC12311776; doi:10.1039/d5tb00304k)
Supplement: TB-013-D5TB00304K-s001 [file TB-013-D5TB00304K-s001.pdf]

Supplementary Information (SI) for Journal of Materials Chemistry B.
This journal is © The Royal Society of Chemistry 2025

Supporting Information

Orthogonal Conjugation of Anchoring-Dependent Membrane Active Peptides for Tuning of Liposome Permeability

*Alexandra Iversen†, Johanna Utterström†, Basab Kanti Das, Robert Selegård, Lalit Pramod Khare, Daniel Aili**

Laboratory of Molecular Materials, Division of Biophysics and Bioengineering, Linköping University, 581 83 Linköping, Sweden.

† equal contribution

* Corresponding author: daniel.aili@liu.se

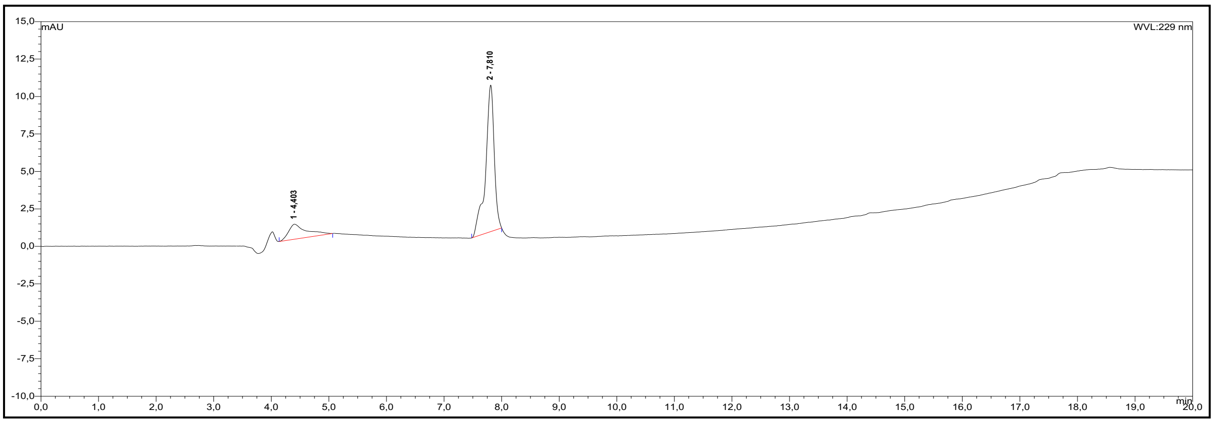

Figure S1. HPLC spectrum of JR2K

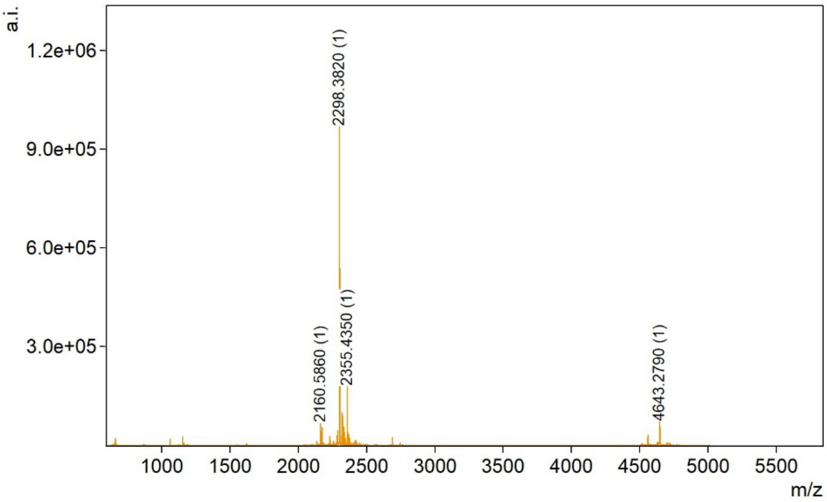

Figure S2. Mass spectrum of JR2K

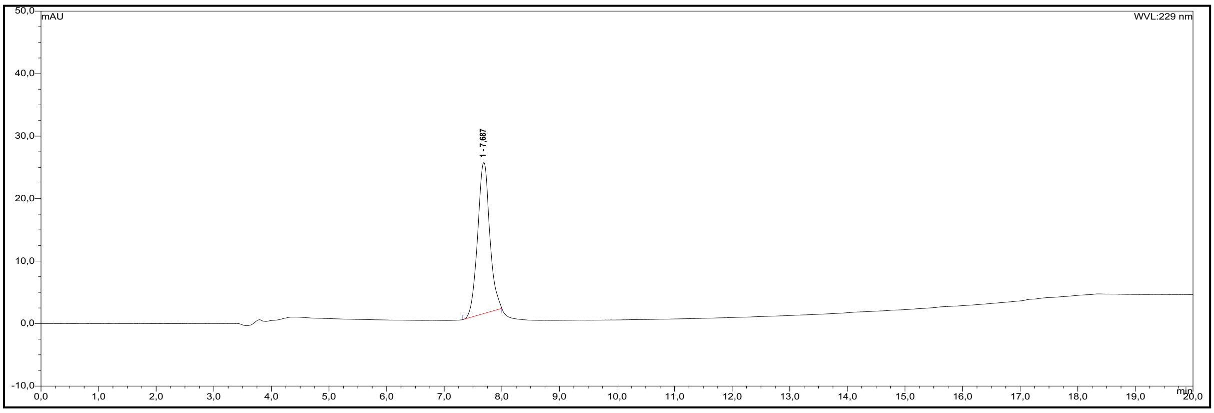

Figure S3. HPLC spectrum of JR2KC

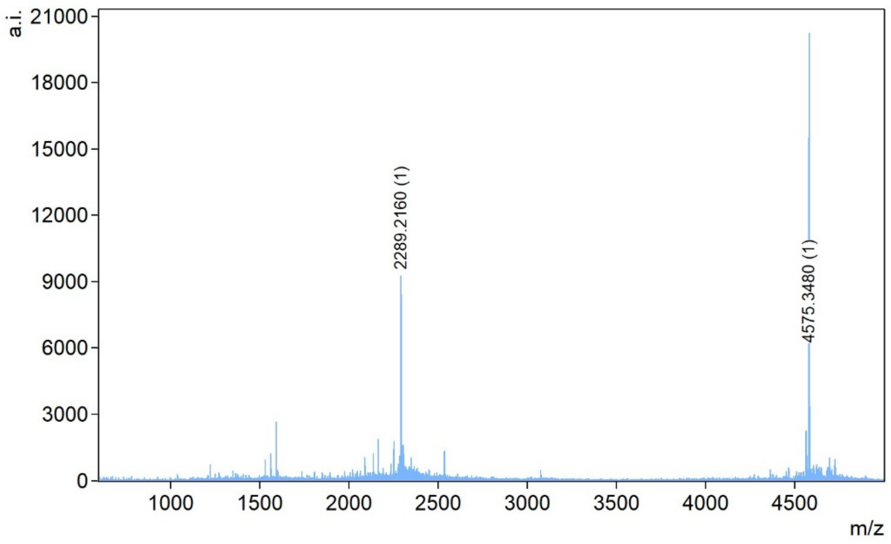

Figure S4. Mass spectrum of JR2KC

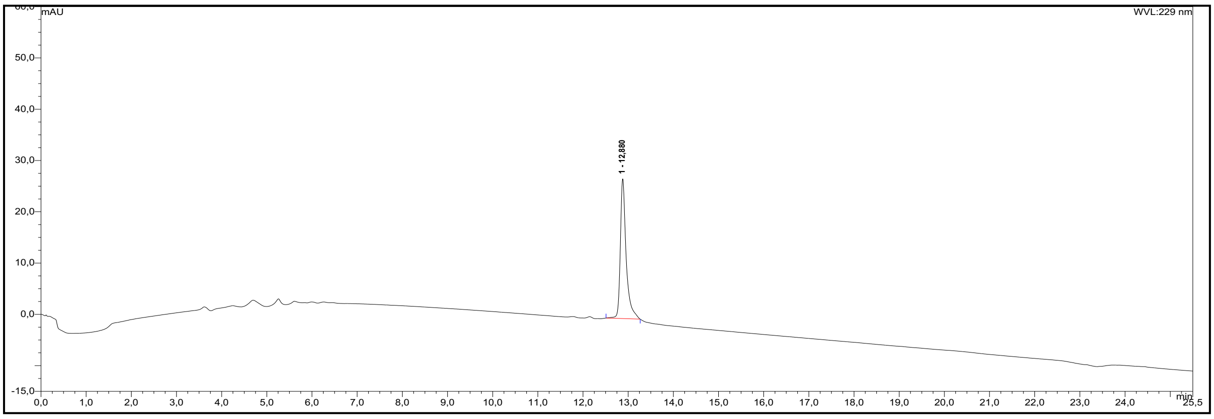

Figure S5. *HPLC spectrum of JR2KC$_{ref}$*

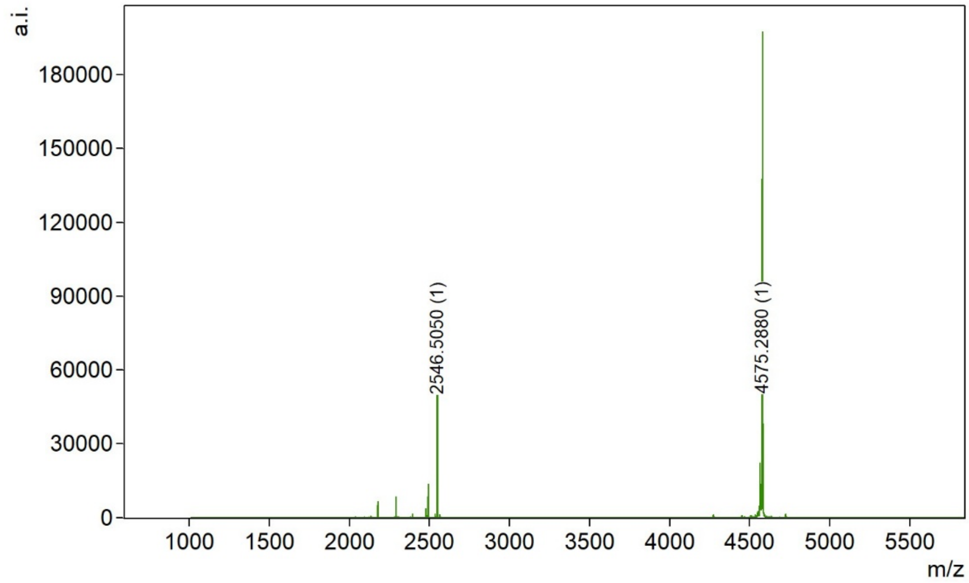

Figure S6. Mass spectrum of $JR2KC_{ref}$

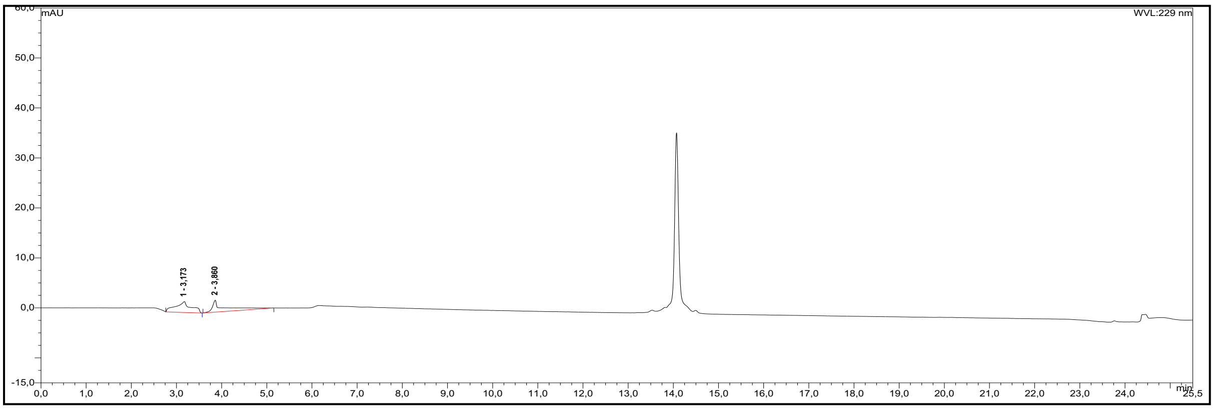

Figure S7. HPLC spectrum of JR2KK-Az

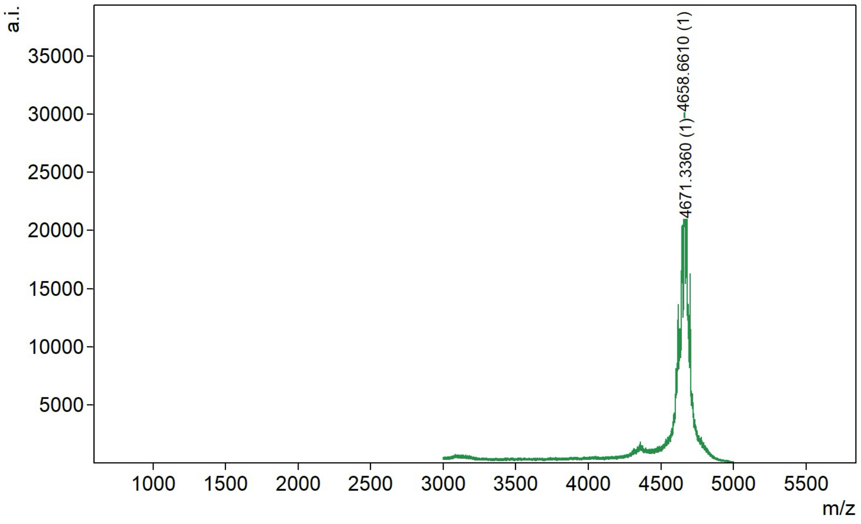

Figure S8. Mass spectrum of JR2KK-Az

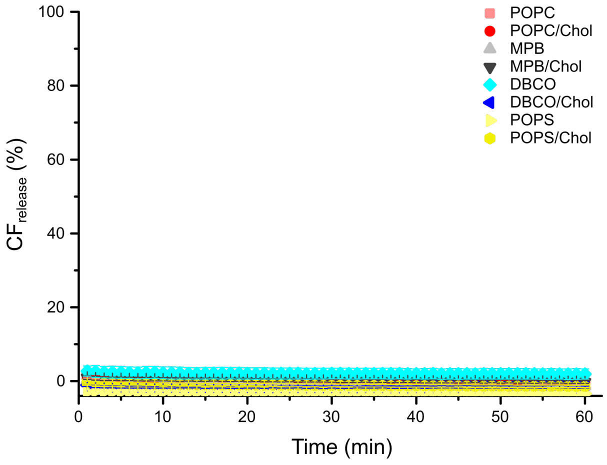

Figure S9. *Total CF release from 40 µM liposomes during 1 h without peptide addition, N = 3.*

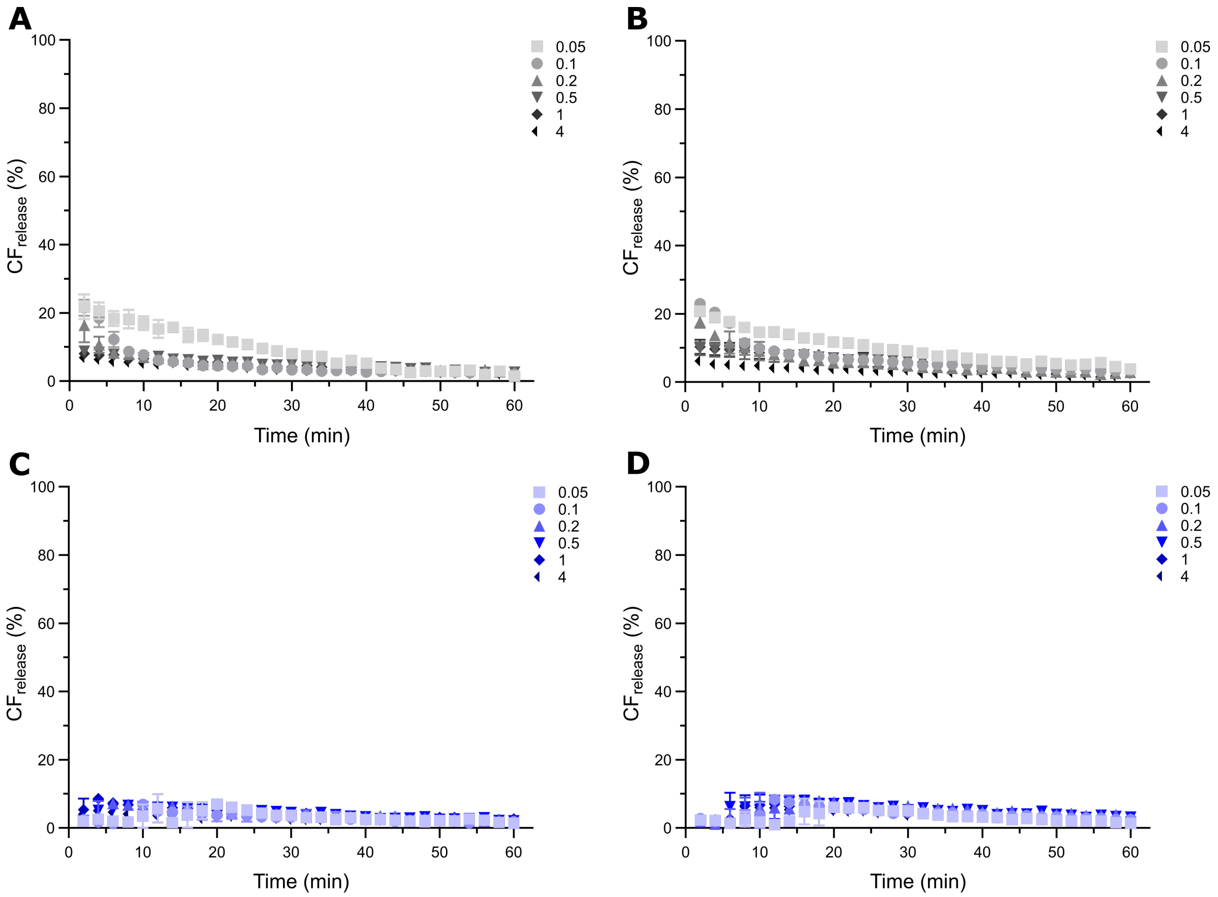

Figure S10. *CF release kinetics during 1 h incubation of (A) JR2KC (0.05 – 4 µM) with 40 µM POPC, (B) JR2KC (0.05 – 4 µM) with 40 µM POPC/Chol, (C) JR2KK-Az (0.05 – 4 µM) with 40 µM POPC and (D) JR2KK-Az (0.05 – 4 µM) with 40 µM POPC/Chol liposomes, N = 3.*

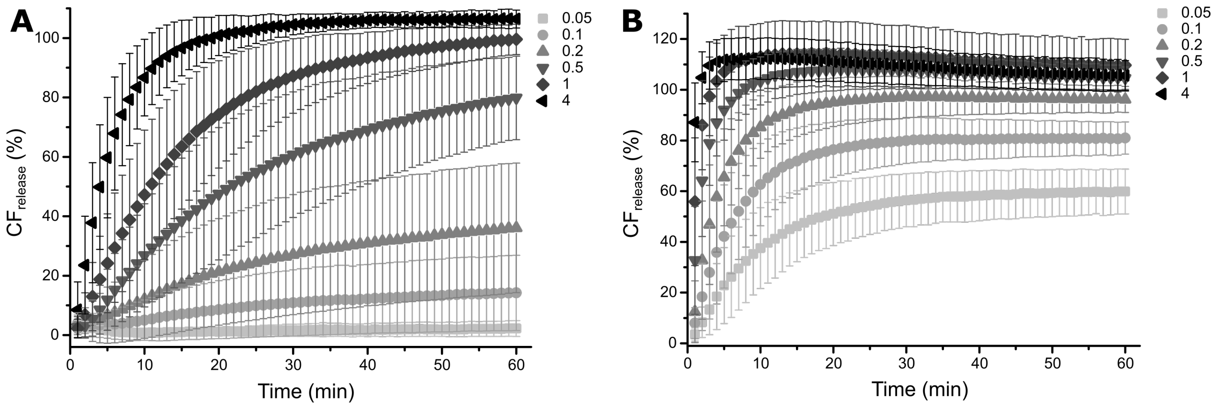

Figure S11. *CF release kinetics during 1 h incubation of JR2KC (0.05 – 4 µM) with 40 µM (A) MPB, N = 10 and (B) MPB/Chol liposomes, N = 8.*

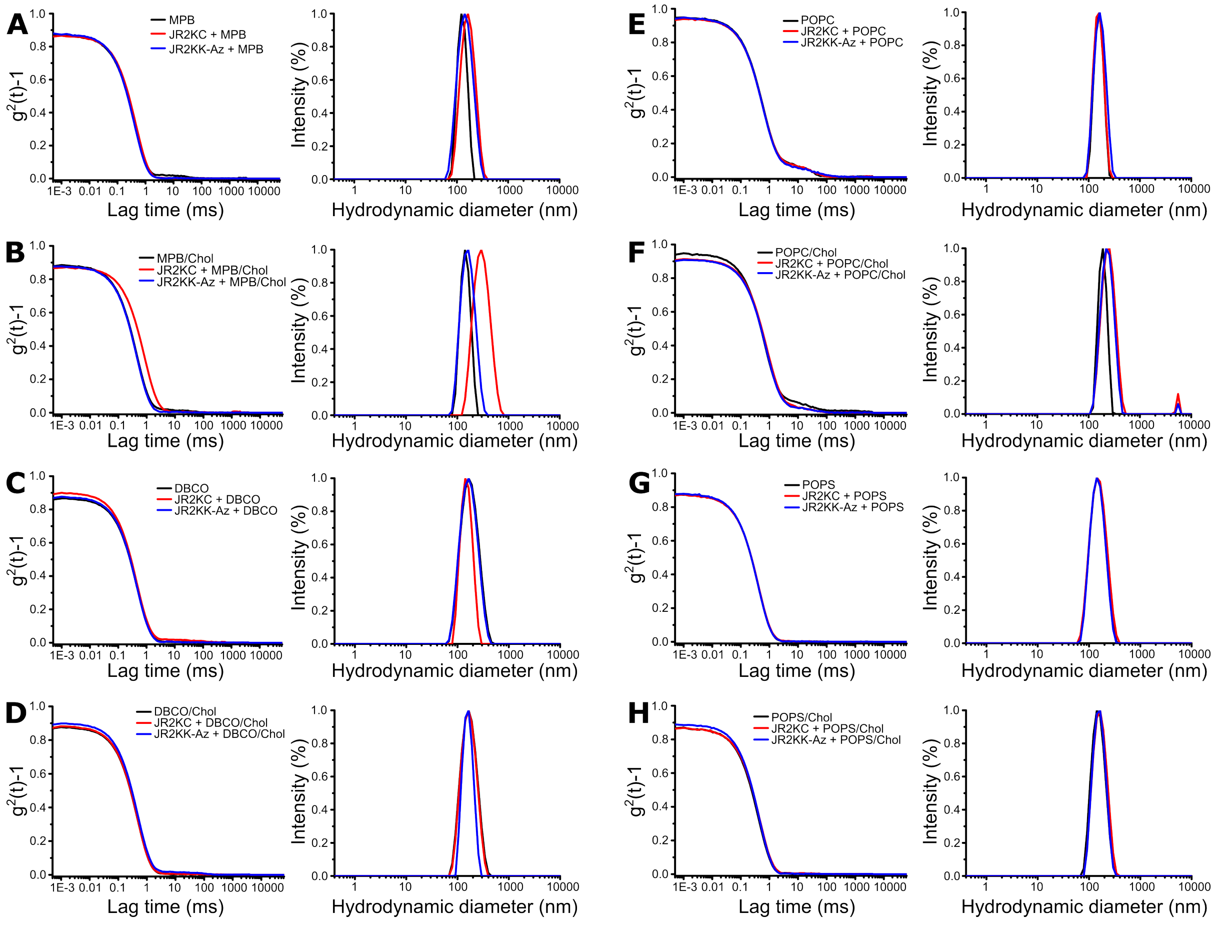

Figure S12. *DLS correlation functions (left) and intensity size distributions (right) of 40 µM liposomes (black), liposomes with 1 µM JR2KC (red), and liposomes with 1 µM JR2KK-Az (blue), recorded 1 h after peptide addition. Lipid compositions according to (A) MPB, (B) MPB/Chol, (C) DBCO (D) DBCO/Chol, (E) POPC, (F) POPC/Chol, (G) POPS and (H) POPS/Chol.*

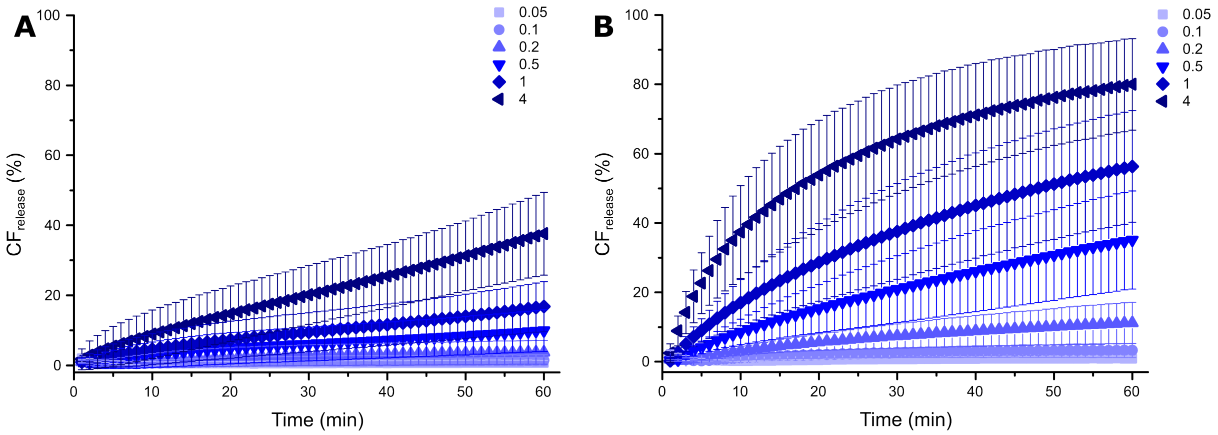

Figure S13. *CF release kinetics during 1 h incubation of JR2KK-Az (0.05 – 4 µM) with 40 µM (A) DBCO, N = 12, and (B) DBCO/Chol liposomes, N = 15.*

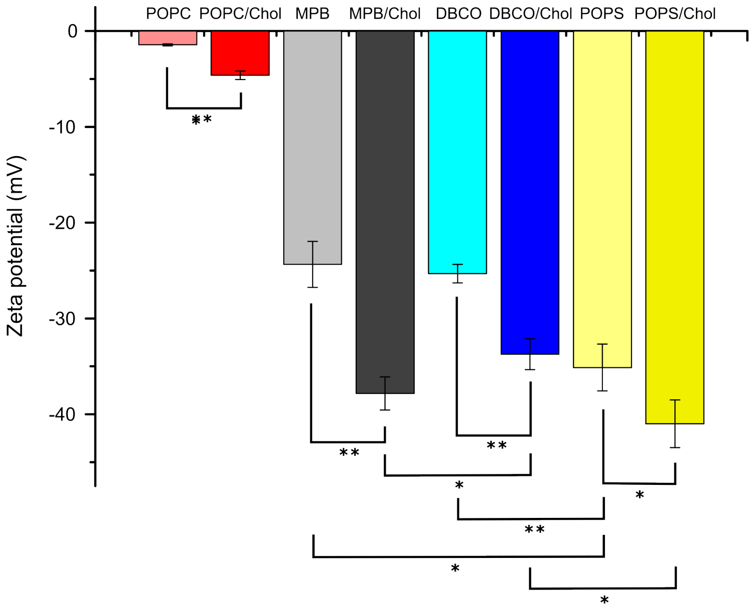

Figure S14. *Zeta potential of the different liposomes used (p-value: * < 0.05, ** < 0.005 and *** < 0.0005).*

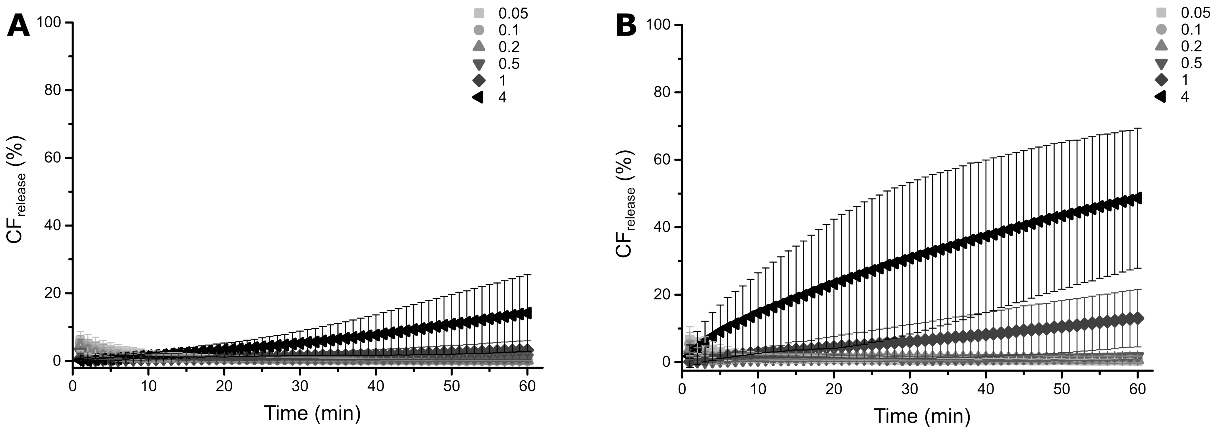

Figure S15. *CF release kinetics during 1 h incubation of JR2KK-Az (0.05 – 4 µM) with 40 µM (A) MPB, N = 11, and (B) MPB/Chol liposomes, N = 8.*

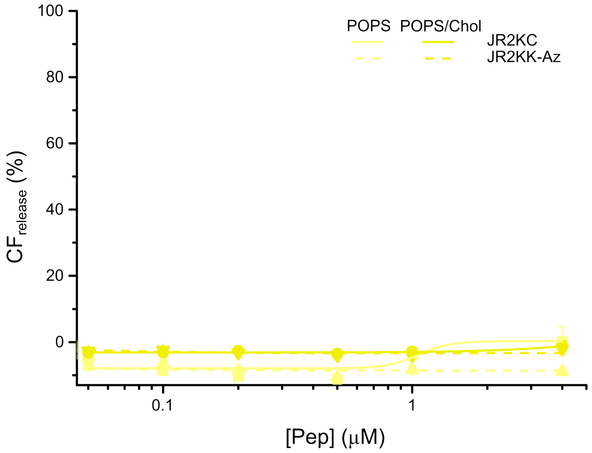

Figure S16. *Total CF release after 1 h incubation with JR2KC (solid) and JR2KK-Az (dashed) on 40 µM POPS-liposomes, with (dark yellow) and without (light yellow) cholesterol, N = 3.*

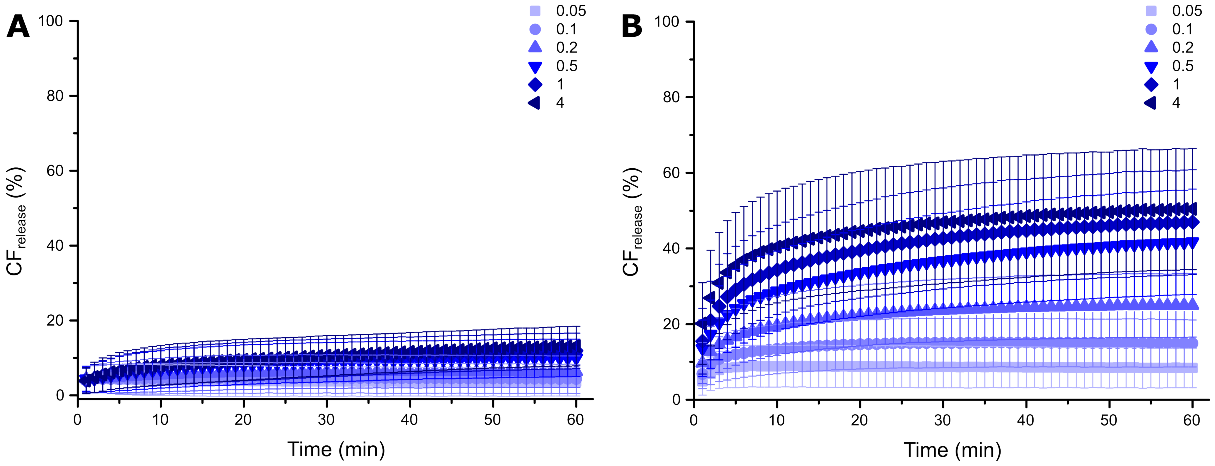

Figure S17. *CF release kinetics during 1 h incubation of JR2KC (0.05 – 4 µM) with 40 µM (A) DBCO, N = 12, and (B) DBCO/Chol liposomes, N = 15.*

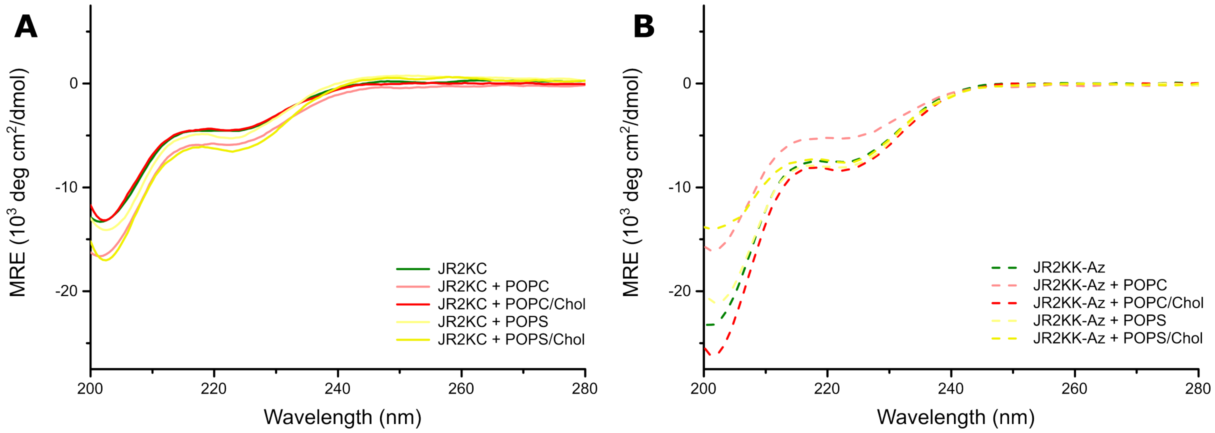

Figure S18. *CD spectra of 30 µM (A) JR2KC (solid) and (B) JR2KK-Az (dashed), alone (green) or incubated for 1 h with 1.2 mM POPC (pink), POPC/Chol (red), POPS (light yellow) and POPS/Chol (dark yellow) liposomes.*

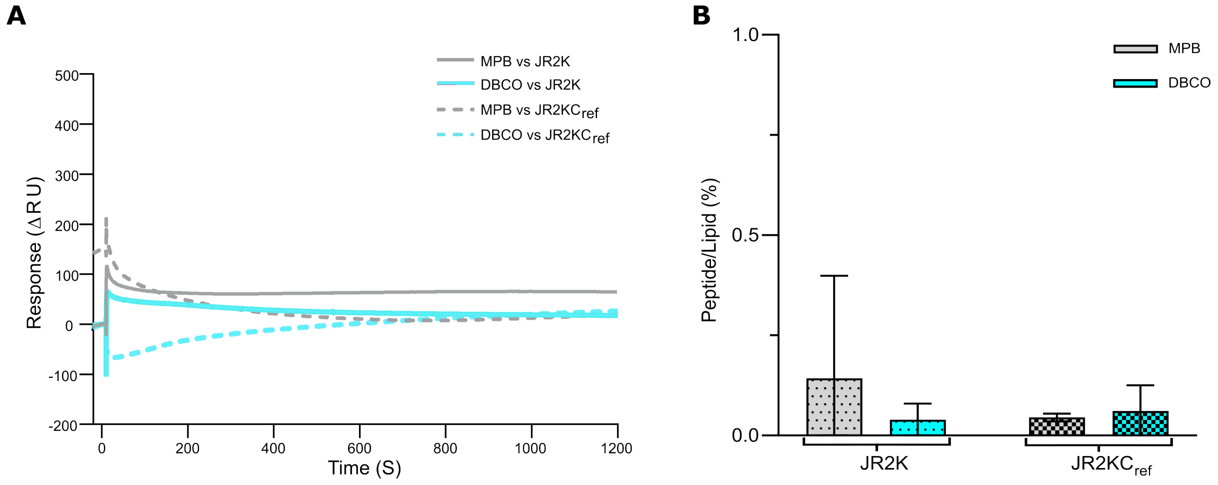

Figure S19. *(A) SPR sensograms showing the conjugation of JR2K and JR2KC$_{ref}$ peptides immobilized 5 mol% MPB/DBCO liposomes. (B) Estimation of the number of peptides conjugated per lipid in 5 mol% MPB/DBCO liposomal systems.*

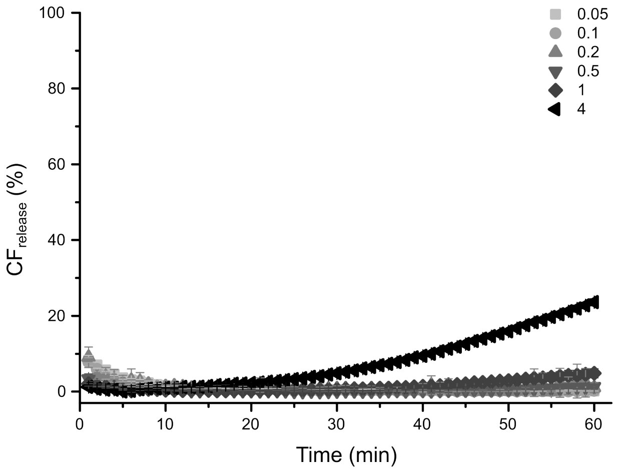

Figure S20. *CF release kinetics during 1 h incubation of JR2KK-Az (0.05 – 4 µM) with 40 µM MPB liposomes (encapsulating CF) and 40 µM DBCO/Chol liposomes (encapsulating PBS).*
